# Characterization of the substitution pattern of cellulose derivatives using carbohydrate-binding modules

**DOI:** 10.1186/s12896-014-0113-9

**Published:** 2014-12-24

**Authors:** Laura von Schantz, Herje Schagerlöf, Eva Nordberg Karlsson, Mats Ohlin

**Affiliations:** Department of Immunotechnology, Lund University, Lund, Sweden; Department of Biochemistry & Structural Biology, Lund University, Lund, Sweden; Department of Biotechnology, Lund University, Lund, Sweden; Present Addresses: Alligator Bioscience AB, Lund, Sweden; Present Addresses: Camurus AB, Lund, Sweden

**Keywords:** Substitution pattern, Methylcellulose, Hydroxypropyl methylcellulose, Application of carbohydrate-binding modules

## Abstract

**Background:**

Derivatized celluloses, such as methylcellulose (MC) and hydroxypropyl methylcellulose (HPMC), are of pharmaceutical importance and extensively employed in tablet matrices. Each batch of derivatized cellulose is thoroughly characterized before utilized in tablet formulations as batch-to-batch differences can affect drug release. The substitution pattern of the derivatized cellulose polymers, i.e. the mode on which the substituent groups are dispersed along the cellulose backbone, can vary from batch-to-batch and is a factor that can influence drug release.

**Results:**

In the present study an analytical approach for the characterization of the substitution pattern of derivatized celluloses is presented, which is based on the use of carbohydrate-binding modules (CBMs) and affinity electrophoresis. CBM4-2 from *Rhodothermus marinus* xylanase 10A is capable of distinguishing between batches of derivatized cellulose with different substitution patterns. This is demonstrated by a higher migration retardation of the CBM in acrylamide gels containing batches of MC and HPMC with a more heterogeneous distribution pattern.

**Conclusions:**

We conclude that CBMs have the potential to characterize the substitution pattern of cellulose derivatives and anticipate that with use of CBMs with a very selective recognition capacity it will be possible to more extensively characterize and standardize important carbohydrates used for instance in tablet formulation.

## Background

**Figure 1:**
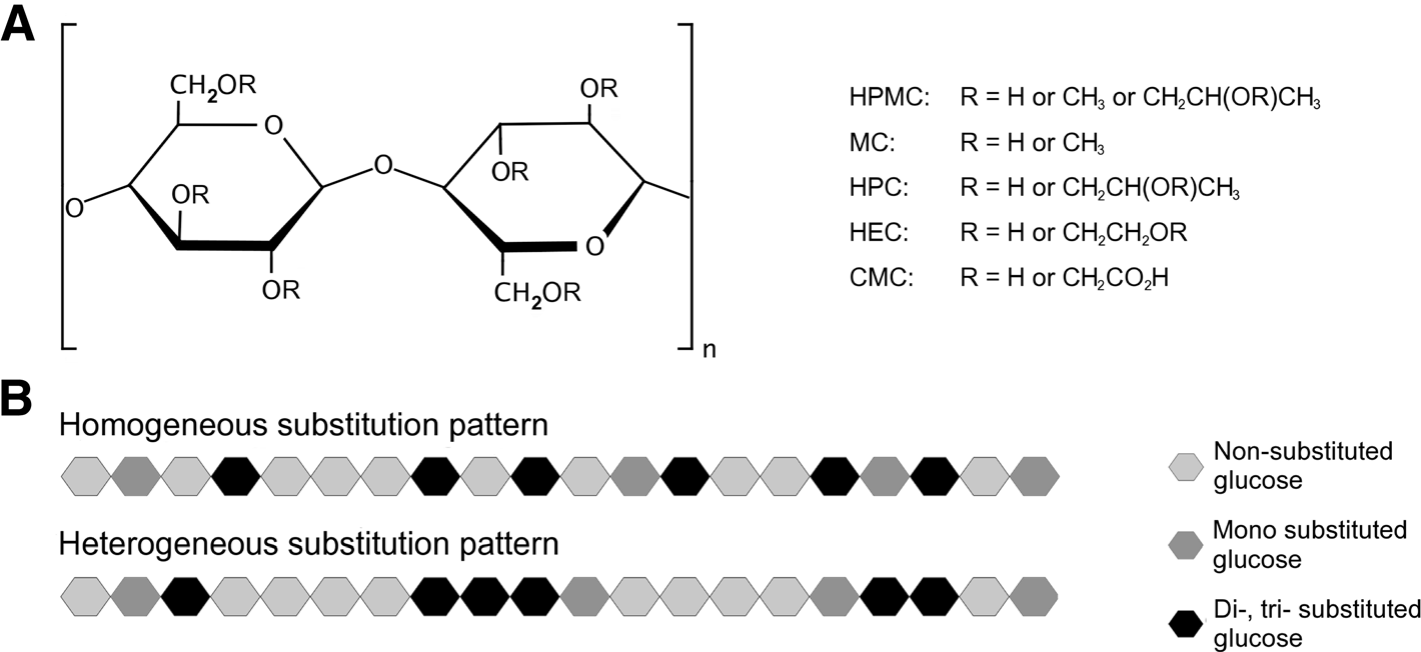
**Chemical structure of different celluloses derivatives. A**. Cellulose consists of glucose saccharides connected through glycosidic 1–4 bonds. In the generation of cellulose derivatives hydrogen atoms (annotated as R) on three hydroxyl groups on each glucose unit are susceptible to chemical derivatization. The possible substitutions for hydroxypropyl methylcellulose (HPMC), methylcellulose (MC), hydroxypropyl cellulose (HPC), hydroxyethyl cellulose (HEC) and carboxymethyl cellulose (CMC) are shown. **B**. Schematic illustration of substitution patterns in homogenously and heterogeneously substituted cellulose.

Cellulose is the most abundant natural biopolymer on earth [1]. It is primarily used to produce paper but as it is renewable and abundant, cellulose is also an attractive raw material for various industrial uses. However, native cellulose from plants and wood has a crystalline structure that complicates its use in bioprocesses. It consists of glucose saccharides bonded through glycosidic 1–4 β-bonds (Figure 1) to form linear polymer chains that have very strong inter-chain attractions making the complex insoluble [2]. Chemical modification of cellulose resulting in the incorporation of functional groups at the free hydroxyl groups along the cellulose chains improves the solubility of the carbohydrate [3].

Derivatized cellulose is used in a wide range of industries and included in food, textile, healthcare and personal care products. In the pharmaceutical industry derivatized celluloses are often used as excipients [1-3]. A common formulation is mixing of the active substance with a polymer in a compressed tablet that upon hydration swells into a gel from which a controlled, prolonged delivery of the drug occurs [4,5]. Hydroxypropyl methylcellulose (HPMC) is the most frequently used polymer in such tablets [6], but other cellulose ethers with different patterns of substitutions are also commonly used (Figure 1), such as methylcellulose (MC), hydroxypropyl cellulose (HPC), hydroxyethyl cellulose (HEC) and carboxymethyl cellulose (CMC) [1,5,6].

The physicochemical properties of the derivatized polymer chains have been shown to affect their behavior and applicability as excipients in tablets [4]. An important factor affecting polymer behavior and drug release has been shown to be the average molecular weight that e.g. influences the viscosity of the dissolving tablets [7-10]. Other critical factors observed are the type and degree of substitution as well as the average number and position of the substituents in a single glucose unit [11-14]. In studies by Viridén et al. [15,16], tablets produced from seven different commercial HPMC batches were investigated. The batches had the same substitution grade and viscosity but behaved differently with respect to their capacity for gelation and polymer release and it was concluded that these differences were correlated to the substitution pattern of the HPMC batches. In a later study, Viridén et al. also showed that the drug release mechanism in HPMC tablets is controlled to a greater extent by erosion if the HPMC has a substitution pattern that is homogeneous, while it is largely controlled by diffusion if the HPMC has a more heterogeneous substitution pattern [17]. Thus it is important to be able to characterize the raw material used as excipient in tablets in order to minimize batch-to-batch variation in the tablet production process.

Though methods for determining the localization of substitutions within a glucose unit are well established [18-20], the characterization of the substitution pattern along the glucose backbone is more challenging and often involves a combination of several techniques. Many of the approaches require partial hydrolysis of the derivatized cellulose chains, which can be performed either randomly, by chemical strategies [21], or by using hydrolyzing enzymes that cleave the chains at specific regions [22]. After partial hydrolysis, the chemical structures of the resulting oligomers are often analyzed using methods such as mass spectrometry (MS) or nuclear magnetic resonance NMR [23]. MS analyses have been performed both with matrix-assisted laser desorption/ionization time of flight (MALDI-TOF) mass spectrometry [13] as well as with electrospray ionization (ESI), with or without separation with liquid chromatography [12,24,25]. Such analyses have in turn been performed either with or without prior isotopic labeling [26,27]. Other strategies differentiate the modified cellulose polymers by use of their cloud point, i.e. the gelation temperature, which partly depends on the substitution pattern [28,29].

Enzyme-aided characterization of cellulose derivatives is an attractive approach as it is based on specific protein-carbohydrate interactions [30]. Other proteins that can specifically recognize natural cellulose are carbohydrate-binding modules (CBMs). CBMs are defined as non-catalytic modules of carbohydrate active enzymes that have a continuous polypeptide chain and a separate fold and display carbohydrate-binding activity [31]. In the present study it was investigated if the specificity in natural and engineered CBMs can be used for characterization of the substitution pattern of cellulose derivatives by affinity electrophoresis (AE). Two natural CBMs with reported ability to bind to cellulose oligomers and regenerated cellulose were used, CBM4-2 [32] and CBM28 [33]. In addition, three mutants derived from CBM4-2 were used, of which A-6 was selected for its binding to Avicel [34], X-2 L110F for its binding to several polysaccharides including β-glucan and cellulose oligomers [35], and G-4 for its lack of binding to carbohydrates (i.e. it serves as a negative control) [34,36]. The mutants had been engineered for other studies and differ from CBM4-2 by a set of 7 to 9 mutations (Table 1) mostly found in the binding cleft. Also, because it is known that CBMs arranged in tandem often display a higher apparent affinity (avidity) for their target [37], a construct consisting of two connected CBM4-2 modules was included in the study. Altogether, these CBMs represent a collection of modules with different properties likely to represent different patterns of derivatized cellulose recognition.

**Table 1:** **Amino acid differences existing between CBM4-2 and mutants thereof**

	**67**	**68**	**69**	**70**	**71**	**72**	**73**	**74**	**75**	**76**	**77**	**78**	**108**	**109**	**110**	**111**	**112**	**113**	**114**	**115**	**116**	**117**	**118**	**119**	**120**	**140**	**141**	**142**	**143**	**144**	**145**	**146**	**147**	**148**	**149**	**150**	mutations outside the binding cleft
**CBM4-2**	**N**	**P**	**W**	**D**	**I**	**E**	**A**	**T**	**A**	**F**	**P**	**V**	**Q**	**S**	**F**	**Q**	**E**	**Y**	**G**	**R**	**L**	**H**	**E**	**Q**	**Q**	**V**	**I**	**R**	**A**	**P**	**I**	**H**	**F**	**G**	**Y**	**A**	
**A-6**	**.**	**.**	**.**	**.**	**.**	**Q**	**.**	**.**	**.**	**H**	**.**	**.**	**.**	**.**	**Y**	**.**	**D**	**.**	**.**	**.**	**.**	**L**	**Q**	**.**	**.**	**.**	**.**	**.**	**.**	**.**	**.**	**.**	**.**	**.**	**L**	**.**	
**X-2 L110F**	**.**	**.**	**F**	**N**	**.**	**Q**	**.**	**.**	**.**	**L**	**.**	**.**	**.**	**.**	**.**	**D**	**.**	**.**	**.**	**.**	**.**	**.**	**E**	**.**	**.**	**.**	**.**	**.**	**.**	**.**	**.**	**.**	**.**	**.**	**.**	**.**	**W91R**
**G-4**	**.**	**.**	**F**	**.**	**.**	**D**	**.**	**.**	**.**	**L**	**.**	**.**	**.**	**.**	**L**	**E**	**H**	**.**	**.**	**.**	**.**	**F**	**.**	**.**	**.**	**.**	**.**	**.**	**.**	**.**	**.**	**Y**	**.**	**.**	**.**	**.**	**E138A**

Through these studies we are able to conclude that CBMs have the potential to discriminate between heterogeneously and homogeneously derivatized celluloses and envisage that such modules can be employed in the characterization of the substitution pattern of cellulose derivatives to be utilized for example in tablet formulations.

## Results

Migration retardation of a set of CBMs was investigated by AE to evaluate if CBMs can distinguish between derivatized celluloses with a homogenous substitution pattern, i.e. randomly dispersed, from those with a heterogeneous substitution pattern, i.e. more clustered substitutions.

### CBM4-2 and X-2 L110F distinguish between batches of MC with different substitution patterns

**Figure 2:**
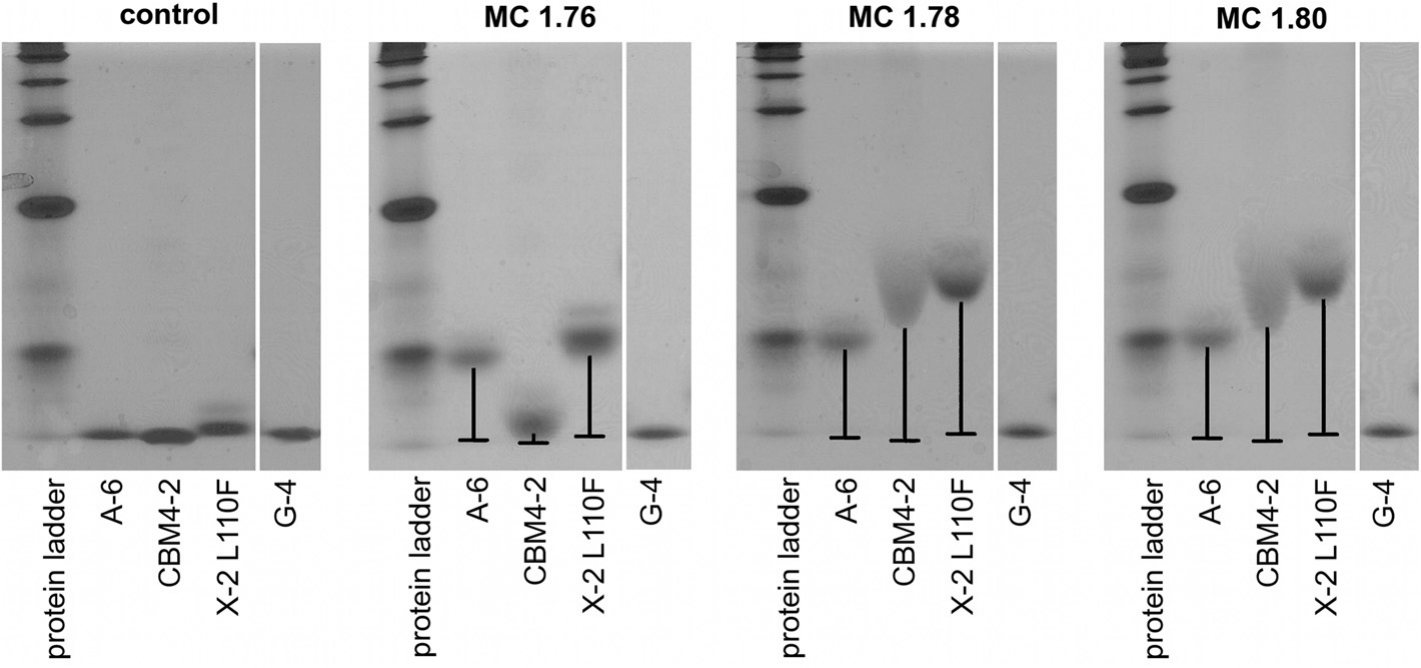
**Affinity electrophoresis with three methylcellulose batches.** The three batches have similar degree of substitution (1.76, 1.78 and 1.80 and denoted accordingly) however their substitution pattern differs. MC 1.76 is more homogeneously substituted while MC 1.78 and MC 1.80 have substitution patterns that are more heterogeneous. Three CBMs and a negative control (G-4) were run on native acrylamide gels containing one of each methylcellulose batch. CBM4-2 is more retained in the gels containing MC 1.78 or MC 1.80 compared to MC 1.76. A-6 and X-2 L110F are less discriminating and are retained in all gels containing methylcellulose. None of the CBMs were retained in the control gel containing no methylcellulose.

Non-denaturing electrophoresis demonstrated that CBM4-2 was retained more in the gels containing the heterogeneously substituted MC 1.78 or MC 1.80 than in the gel with homogeneously substituted MC 1.76 (Figure 2). CBM G-4, a carbohydrate non-binding negative control variant of CBM4-2, was as expected not retained in any of the gels. Of the other CBMs tested, A-6 and X-2 L110F were retained in all of the gels containing MC. X-2 L110F is retained more in the gels containing MC 1.80 and MC 1.78 than MC 1.76 although the difference in mobility is not as apparent as for CBM4-2. In contrast, A-6 is not able to make a distinction between the different MCs illustrating that by using different CBMs it is possible to get different views of the MCs.

### CBM4-2 distinguishes between HPMC batches with different substitution pattern

**Figure 3:**
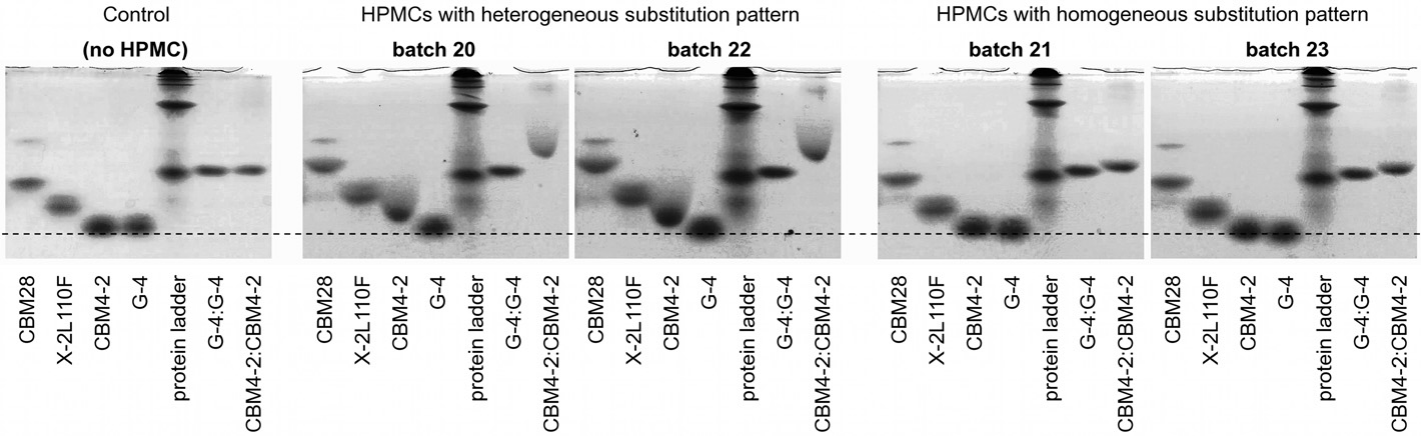
**Affinity electrophoresis with four hydroxypropyl methylcellulose batches.** Two of the batches (20 and 22) are known to have a heterogeneous substitution pattern and the other two (21 and 23) a homogeneous substitution pattern. Three CBMs (CBM28, X-2 L110F and CBM4-2) and a negative control (G-4) were run in 17% acrylamide gels containing no or 1 mg/ml HPMC. Also a tandem CBM construct of CBM4-2:CBM4-2 together with a tandem negative control (G-4:G-4) were included in the assay. The retardation of the CBMs is modest but differences in mobility can be seen for CBM4-2 and CBM28 in gels containing HPMC of heterogeneous substitution pattern as compared to in gels with HPMC of homogeneous substitution pattern. The difference in retardation is more distinct for the tandem CBM4-2 construct.

Gels containing four types of HPMCs were also analyzed for their ability to interact with the CBMs. Of the four different batches included, two of the HPMCs are classified as homogeneously substituted and the other as heterogeneously substituted. In this experiment the CBMs investigated were a negative control (G-4), CBM4-2, X-2 L110F and CBM28 from *B. akibai* Cel5A. The retardations were modest yet a distinction was observed for batches with a heterogeneous versus a homogeneous substitution pattern (Figure 3). In gels containing heterogeneously substituted HPMC, CBM4-2 was retained and the protein band had a trailing shape. In contrast, in gels containing HPMC with a homogeneous substitution pattern, CBM4-2 was hardly retained at all.

### Tandem CBM4-2 construct showed improved interaction with derivatized celluloses

In an effort to achieve better retardation a tandem CBM construct with possible higher apparent affinity to the cellulose derivatives due to avidity effects was generated. CBM4-2 was selected for this experiment as it appeared to be the CBM that is best capable of making a distinction between homogeneously and heterogeneously derivatized cellulose. The tandem construct consisted therefore of two CBM4-2 molecules linked together through a polypeptide linker. Indeed the difference in mobility for the tandem CBM in gels containing homogenously versus heterogeneously derivatized HPMC was more apparent than for the single CBM (Figure 3). It is thus possible to through engineering devise CBM constructs with enhanced ability to distinguish between batches of derivatized cellulose that carry either evenly or clustered distribution of substituents along the glucosyl backbone.

## Discussion

### Proof-of-concept - CBM have the potential to characterize cellulose derivatives

Cellulases are commonly employed for the characterization of derivatized celluloses with respect to their substitution pattern. Many of the enzymes used, such as Cel5A, Cel7B and Cel45 from *Trichoderma reseei* [15,30]*,* EGII from *Trichoderma longibrachiatum* [16], Cel5A from *Bacillus agaradhaerens* [24,30,38] or Cel5A from *Humicola insolens* [30] contain one or more carbohydrate-binding modules linked to either the C- or N-terminus of the catalytic module. The goal of this work was to investigate if CBMs on their own have the ability to make a distinction in the distribution pattern of cellulose derivatives based on their substitution pattern.

In this proof-of-concept study we show that CBMs indeed have the potential to be utilized in the characterization of chemically modified celluloses and can, due to the selective binding affinity displayed by the individual CBM, be used as tools to make a distinction between homogeneously and heterogeneously substituted MC and HPMC. The analysis was carried out using AE that provided a simple readout system. More extensive migration retardation of some CBMs, in particular CBM4-2, was observed in gels containing heterogeneously substituted cellulose derivatives. The molecular rationale for this behavior is not fully understood. The batches of cellulose derivatives with heterogeneous dispersed substituents might carry more binding epitopes and/or such of a nature that can create stronger binding affecting the overall CBM-cellulose polymer interaction, reflected in an increased retardation in the AE gels.

### Visualization of CBM – carbohydrate interactions with affinity electrophoresis provides a rapid and easy read-out

Affinity gel electrophoresis is a technique that combines the visual simplicity of normal native gel electrophoresis with the specific separation of affinity interactions between various macromolecules. It has been widely used for the binding analysis of various interactions including those between antibody-hapten [39], lectin-glycoprotein [40] and between proteins and nucleic acids [41]. In 1977 AE was used to analyze the binding of a lectin against a soluble polysaccharide [42] and following the discovery of CBMs this protocol was adopted for studying the interaction of CBMs and polysaccharides [43]. Today AE is one of the most applied methods for rapid characterization of CBMs with unknown functions or for determining the role of a given amino acid after mutation [44]. In this study we show that this method not only provides information of the CBM but can also be used for the characterization of the polysaccharides. More specifically, the use of AE together with for example CBM4-2 has the potential to function as an initial screening method for the characterization of the substitution pattern of cellulose derivatives.

### Future prospects

To establish a more detailed analysis of the substituent patterns of derivatized celluloses there are multiple possibilities to exploit in terms of a CBM/AE-based assay. An assay of this type may firstly rely on multiple readouts based not only on changes in CBMs’ retardation at a fixed carbohydrate gel content but also on their migration behavior at different concentrations of the derivatized carbohydrate. The second important window of opportunity is to be found in our ability to expand the set of CBMs to include an even more selective binding capacity and to create a multiplexed assay employing multiple CBMs. Such CBM candidates may potentially be of natural origin and found linked to cellulases, but new CBMs can also be obtained through engineering, specifically designed to suit this application. Direct evolution and rational design are powerful approaches for the fine-tuning of protein properties, and a combination of the use of combinatorial libraries and phage-display technology has been successful for the generation of engineered CBMs [34] toward other carbohydrate polymers including xylans [45] and different molecular forms of xyloglucan [46,47]. In the present study CBMs engineered for such natural substrates were included but were unable or inefficient in their ability to discriminate between differentially substituted forms of cellulose. These results were not unexpected since the engineered CBM had not been specifically designed for this application but instead for recognition of other carbohydrate targets. In a future assay one could consider simultaneous use of CBMs that not only represent binders that discriminate substitutions, but also CBMs that specifically interact with these chemical groups, permitting a more complete mapping of the derivatized celluloses. We envisage that engineering of modules with predetermined tolerance for substitutions will be a valuable approach in the implementation of an assay for characterization of cellulose derivatives. It is expected, based on past experience on evolution of CBM binding properties [34,45,46,48], that an appropriately designed phage-display selection process will be able to identify variants from combinatorial libraries that are able to differentiate between differently substituted celluloses. Such engineering will improve assay functionality over the current proof-of-concept study that employed non-optimized CBMs only.

## Conclusion

In conclusion, this study demonstrates that some CBMs have the ability to discriminate between batches of cellulose derivatives with differences in substitution homogeneity. We anticipate that CBMs with better capacity to characterize carbohydrate polymers can be identified from natural sources or created *in vitro* and that such modules can be implemented in a non-degrading screening assay that can complement today’s standard analysis for use in quality control of batches of industrial cellulose derivatives.

## Methods

### Carbohydrate-binding modules

CBM4-2 from *Rhodothermus marinus* xylanase 10A and evolved CBM X-2 L110F [35] were produced in *E. coli* T7 Express (New England Biolabs, Ipswich, England) and purified using affinity chromatography columns on a Ni-NTA resin (Qiagen, Hilden, Germany) as previously described [35]. A codon-optimized gene (synthesized by GeneArt, Life Technologies, Carlsbad, CA, USA) coding for amino acids 571–762 of *Bacillus akibai* (also known as *Bacillus sp.* 1139) cellulase 5A, representing CBM28 [33], carried in the pET22b(+) vector (Novagen, Madison, WI, USA) was transformed into *E. coli* T7 Express. Protein production was made in 200 ml 2 x Yeast Tryptone broth supplemented with 100 μg/ml carbenicillin. The bacteria were cultivated at 37°C with shaking (210 rpm) until OD_600 nm_ reached 0.4. Protein production was induced by addition of 0.5 mM isopropyl-β-D-thiogalactopyranoside followed by a further cultivation at 30°C with shaking (210 rpm) for 4 hours. A-6, which was evolved from CBM4-2 and selected, using phage-display, for its ability to recognize Avicel [34], and G-4, which was also evolved from CBM4-2 but has no carbohydrate-binding activity and is used as negative control [34,36], were produced in *E. coli* BL21(λDE3) as previously described [34].

In an effort to create proteins with stronger binding to cellulose derivatives, we constructed and produced proteins consisting of two CBMs fused together via a peptide linker. Codon-optimized genes encoding such in tandem coupled CBMs carried in the pET22b(+) vector (Novagen) were purchased from GeneArt. The CBMs fused in tandem were produced in *E. coli* T7 Express as described above.

### Cellulose derivatives

Three methylcelluloses were used in this study: MC 1.76 (viscosity type 4 cP), MC 1.78 (viscosity type 15 cP) and MC 1.80 (viscosity type 1500) from Shin-Etsu Chemical Co. (Tokyo, Japan) with similar degree of substitution (DS 1.76, 1.78 and 1.80, respectively) but with different molecular weights (20, 40 respectively 200 kDa) as analyzed by Melander et al. [49], Cohen et al. [24] and Fitzpatrick et al. [23]. The substitution patterns of the MCs have previously been characterized. MC 1.76 is more homogeneously substituted while MC 1.78 and MC 1.80 are more heterogeneously substituted [24]. Four hydroxypropyl methylcellulose (HPMC) batches were also analyzed. Of the HPMC used, batches 20 and 22 were from Dow Wolff Cellulosics (Bomlitz, Germany) and 21 and 23 from Shin-Etsu Chemical Co. All four batches had a similar degree of substitution and molecular weight but batches 21 and 23 were less susceptible to enzymatic degradation, performed with Celluclast (Novozymes A/S, Bagsvaerd, Denmark) and have therefore been classified as homogeneously substituted. Batches 20 and 22 on the other hand were more easily degraded thus classified as carrying a more heterogeneous substitution pattern.

### Affinity electrophoresis

Affinity electrophoresis was used to determine the ability of soluble, charged, mobile CBMs to interact with largely immobile carbohydrates (and thus to become less mobile) under the influence of an electric field. Polyacrylamide gels for analysis of native proteins were cast according to the Ornstein-Davis method [50,51] with the important modification of the addition of polysaccharides. The retardation of the CBM (3 μg/lane) was investigated using gels containing MC or HPMC at a concentration of 3 and 1 mg/ml, respectively. The gels were polymerized in cassettes (Life Technologies) at an acrylamide/bisacrylamide concentration of 12.5/0.42% for gels containing MC and 17.5/0.58% for gels containing HPMC and run in a minigel system for 1 hour and 45 minutes. Staining of protein was done with SimplyBlue (Life Technologies) for 20 minutes, followed by destaining in water according to the manufacturer’s instructions.

## Abbreviations

CBM: Carbohydrate-binding module
AE: Affinity electrophoresis
HPMC: Hydroxypropyl methyl cellulose
MC: Methyl cellulose
HPC: Hydroxypropyl cellulose
HEC: Hydroxyethyl cellulose
CMC: Carboxymethyl cellulose
MS: Mass spectrometry
NMR: Nuclear magnetic resonance
MALDI-ToF: Matrix-assisted laser desorption/ionizaion time of flight)
ESI: Electrospray ionization
